# Extensive Subcutaneous Emphysema and Pneumomediastinum after Ecstasy Ingestion

**DOI:** 10.1155/2013/795867

**Published:** 2013-09-25

**Authors:** A. Gungadeen, J. Moor

**Affiliations:** ENT Department, City Hospitals Sunderland NHS Foundation Trust, Kayll Road, Sunderland SR4 7TP, UK

## Abstract

*Objective*. To present a rare case of extensive subcutaneous emphysema and spontaneous pneumomediastinum following ingestion of Ecstasy in a young adult. We also review the relevant literature and discuss how this case supplements it. *Case Report*. We report a case of a 19-year-old man with a history of painless neck and chest swelling, and no chest pain or breathlessness, after consuming Ecstasy tablets. Radiological imaging showed evidence of pneumomediastinum and extensive subcutaneous emphysema. The patient remained well under observation and his symptoms improved with conservative management. *Conclusions*. Subcutaneous emphysema and pneumomediastinum after Ecstasy ingestion is uncommon. Cases are often referred to the otolaryngologist as they can present with neck and throat symptoms. Our case showed that the severity of symptoms may not correlate with severity of the anatomical abnormality and that pneumomediastinum should be suspected in Ecstasy users who present with neck swelling despite the absence of chest symptoms. Although all cases reported so far resolved with conservative management, it is important to perform simple investigations to exclude coexisting serious pathology.

## 1. Introduction

The occurrence of subcutaneous emphysema and pneumomediastinum is rare following ingestion of illicit drugs. Ecstasy is a street drug with the active substance 3,4-methylenedioxymethamphetamine (MDMA). It is one of the most commonly used illegal drugs in the UK, with up to two million tablets consumed every week [1]. In this paper, we describe a case of extensive subcutaneous emphysema and spontaneous pneumomediastinum following use of Ecstasy. We also review the relevant literature and discuss how this case supplements it.

## 2. Case Presentation

A 19-year-old otherwise healthy man presented to Accident and Emergency, on his mother's request, after having awoken four hours earlier with a feeling of “crushed ice” under the skin of his neck, chest, and back. He had no dyspnoea, dysphagia, neck pain, or voice change and had very slight odynophagia. He had drunk heavily the night before and alleged that he had taken 12 Ecstasy tablets during the course of that evening. He denied any history of recent trauma or vigorous physical exertion. He had had no recent episodes of coughing or vomiting. He was on no regular medication, but frequently used street drugs and alcohol.

On examination, he was alert, with no obvious respiratory compromise. Blood pressure was 139/73, pulse was 93; respiratory rate was 16 per minute, temperature was normal, and oxygen saturation was 99%. Subcutaneous emphysema was easily palpable over an extremely wide area of the neck and chest. There was no evidence of external trauma. Cardiorespiratory and abdominal examinations were otherwise unremarkable. Examination of the larynx using a flexible fibreoptic nasendoscopy was normal.

**Figure 1:**
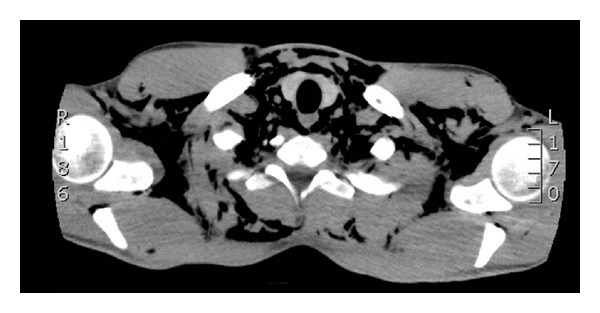
Axial CT image showing gross subcutaneous emphysema.

**Figure 2:**
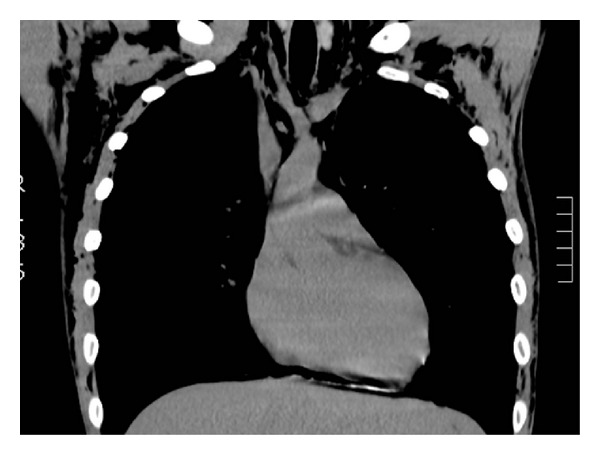
Coronal CT image showing pneumomediastinum and subcutaneous emphysema.

Routine blood tests were normal. Arterial blood gases on room air showed a mild respiratory alkalosis (pH 7.48, pCO_2_ 4.16 kPa, pO_2_ 12.1 kPa, HCO_3_^−^ 25.5 mmol/L). Chest radiograph showed surgical emphysema and no pneumothorax. Computed tomography showed pneumomediastinum and extensive subcutaneous emphysema extending from the mastoid tip to the upper abdominal wall involving several fascial planes; no convincing pneumothorax was identified, and the lungs were clear (see Figures 1 and 2). Water-soluble contrast swallow was unremarkable and showed no evidence of oesophageal perforation.

## 3. Management

The patient was managed conservatively with close observation on the ENT ward. He was kept “nil by mouth” until the next morning, after which he tolerated diet well. He had an uneventful stay and his subcutaneous emphysema gradually improved. He was discharged 48 hours after admission.

## 4. Discussion

The underlying pathogenesis of subcutaneous emphysema and pneumomediastinum after Ecstasy use remains unclear. No direct pharmacological link has been demonstrated and no association has been seen between amounts of drug used and the severity of cases, although it is difficult to ascertain concentrations of Ecstasy used in different cases. It has been suggested that spontaneous pneumomediastinum arises from perivascular alveolar rupture and subsequent escape of air through the vascular sheaths into the mediastinum, and thereafter to the fascial planes of the neck, as the air follows the path of least resistance [2]. Perivascular alveolar rupture can be caused by sudden increases in the bronchovascular pressure gradient, through an increase in alveolar pressure such as in mechanical ventilation and Valsalva manoeuvre, or a decrease in pulmonary interstitial pressure such as in asthma and bronchiolitis [3].

Subcutaneous emphysema and pneumomediastinum after inhaled drugs such as cocaine and marijuana is a more common phenomenon than after ingested substances [2, 4]. This can be explained by the barotrauma induced by the ritualistic Valsalva manoeuvre used to enhance the effect of drugs being snorted or smoked [4].

However, ingestion of substances such as Ecstasy does not involve similar barotrauma. Several theories have been proposed for the presence of subcutaneous emphysema and pneumomediastinum in these cases. Ecstasy is commonly used at “rave” parties where participants engage in long periods of strenuous dancing. These levels of vigorous activity are thought to predispose the Ecstasy users to increased alveolar pressure, and ultimately alveolar rupture [5]. Similarly, prolonged sexual activity has also been linked to increased risk of alveolar rupture in Ecstasy users [6]. 

It has also been suggested that the theory of increased alveolar pressure through vigorous activity might not be the only causative factor. Another possibility that has been proposed is that contaminants in the drug preparations might have unknown pharmacological actions which could predispose to alveolar rupture [7].

In our case, the patient denied any strenuous activity that would predispose him to alveolar rupture. Although this could be due to poor recall as Ecstasy is associated with reduced memory [1], it raises the possibility that there are other undetermined factors that predispose to subcutaneous emphysema and pneumomediastinum.

### 4.1. Clinical Features

All 22 previous cases of ecstasy-related pneumomediastinum reported in the literature presented with either chest pain and/or breathlessness [3, 5–16]. The chest pain is usually pleuritic in nature and has been reported to radiate to the neck [8] or back [8, 10]. Neck swelling corresponding to underlying subcutaneous emphysema is also common [3, 5, 8, 13, 15, 16]. Dysphagia [6, 10], sore throat/odynophagia [3, 5, 9, 16, 17], and dysphonia [7] have also been documented. The time of presentation is variable as patients present several hours to days after consumption of the substance.

Examination shows only subcutaneous emphysema in many patients [3, 5, 6, 8, 13, 16]. Tachycardia and Hamman's sign can also be present [5, 7, 9, 11, 15, 17, 18].

Our case demonstrates that Ecstasy-induced pneumomediastinum can present without chest pain or breathlessness. The patient's presenting symptom was neck swelling despite the gross extent of subcutaneous emphysema and pneumomediastinum, as evident from the imaging performed. This is clinically important as pneumomediastinum should be suspected in Ecstasy users who present with neck swelling despite the absence of chest symptoms. The case also demonstrates that the severity of symptoms may not correlate with the severity of the anatomical abnormality.

### 4.2. Investigations

Electrocardiograms and blood tests in reported cases have been unremarkable [6, 10]. Echocardiogram in one case showed evidence of pneumomediastinum [7]. 

Chest radiographs are usually the initial imaging modality used. They often show subcutaneous emphysema [3, 8, 10, 12] and may suggest pneumomediastinum [7–9, 13–15]. They also reveal any related pneumothoraces [3, 5, 15]. Computed tomography (CT) helps identify and quantify pneumomediastinum and pneumothoraces [5–7, 10, 12].

Gastrografin swallows are also used in many patients and are mostly negative. In one reported case, a small leak was identified in the mid-oesophagus, which was resolved within a few days [8]. Despite the lack of positive findings in most cases, swallow tests can be useful to exclude spontaneous oesophageal rupture.

### 4.3. Treatment

All reported cases of Ecstasy-related subcutaneous emphysema and pneumomediastinum have been managed with conservative measures and remained self-limiting [3, 5–16]. Bed rest and a period of observation whilst “nil by mouth,” followed by gradual reintroduction of a normal diet, have led to successful discharge.

This is consistent with how our patient was treated. An observation period is warranted as it allows clinicians to assess the course of the condition. Clinical evidence of improvement or resolution of symptoms can help reassure patients and clinicians prior to discharge. Worsening of coexisting pneumothoraces can also be potentially identified and treated accordingly. Advice to patients regarding symptoms to watch out for is also essential on discharge.

## 5. Conclusion

Subcutaneous emphysema and pneumomediastinum after Ecstasy ingestion is rare. Cases are often referred to the otolaryngologist as they can present with neck and throat symptoms. Our case showed that the severity of symptoms may not correlate with severity of the anatomical abnormality and that pneumomediastinum should be suspected in Ecstasy users who present with neck swelling despite the absence of chest symptoms. Although all cases reported so far were resolved with conservative management, it is important to investigate the patients to exclude coexisting serious pathology.
